# Increased PD-1 Level in Severe Cervical Injury Is Associated With the Rare Programmed Cell Death 1 (*PDCD1*) rs36084323 A Allele in a Dominant Model

**DOI:** 10.3389/fcimb.2021.587932

**Published:** 2021-07-01

**Authors:** Mauro César da Silva, Fernanda Silva Medeiros, Neila Caroline Henrique da Silva, Larissa Albuquerque Paiva, Fabiana Oliveira dos Santos Gomes, Matheus Costa e Silva, Thailany Thays Gomes, Christina Alves Peixoto, Maria Carolina Valença Rygaard, Maria Luiza Bezerra Menezes, Stefan Welkovic, Eduardo Antônio Donadi, Norma Lucena-Silva

**Affiliations:** ^1^ Laboratory of Immunogenetics, Department of Immunology, Aggeu Magalhães Institute, Oswaldo Cruz Foundation, Recife, Brazil; ^2^ Getúlio Vargas Hospital, Pernambuco Health Department, Recife, Brazil; ^3^ Clinical Immunology Division, Department of Medicine, School of Medicine of Ribeirão Preto, University of São Paulo (USP), Ribeirão Preto, Brazil; ^4^ Laboratory of Molecular Biology, IMIP Hospital, Pediatric Oncology Service, Recife, Brazil; ^5^ Department of Maternal and Child, Faculty of Medical Sciences, University of Pernambuco, Recife, Brazil; ^6^ Integrated Health Center Amaury de Medeiros (CISAM), University of Pernambuco, Recife, Brazil

**Keywords:** PD-1, CIN, HPV, polymorphism, inflammation, cancer

## Abstract

The high-risk oncogenic human papillomavirus (HPV) has developed mechanisms for evasion of the immune system, favoring the persistence of the infection. The chronic inflammation further contributes to the progression of tissue injury to cervical cancer. The programmed cell death protein (PD-1) after contacting with its ligands (PD-L1 and PD-L2) exerts an inhibitory effect on the cellular immune response, maintaining the balance between activation, tolerance, and immune cell-dependent lesion. We evaluated 295 patients exhibiting or not HPV infection, stratified according to the location (injured and adjacent non-injured areas) and severity of the lesion (benign, pre-malignant lesions). Additionally, we investigated the role of the promoter region *PDCD1* -606G>A polymorphism (rs36084323) on the studied variables. PD-1 and *PDCD1* expression were evaluated by immunohistochemistry and qPCR, respectively, and the *PDCD1* polymorphism was evaluated by nucleotide sequencing. Irrespective of the severity of the lesion, PD-1 levels were increased compared to adjacent uninjured areas. Additionally, in cervical intraepithelial neoplasia (CIN) I, the presence of HPV was associated with increased (*P* = 0.0649), whereas in CIN III was associated with decreased (*P* = 0.0148) PD-1 levels, compared to the uninjured area in absence of HPV infection. The *PDCD1* -606A allele was rare in our population (8.7%) and was not associated with the risk for development of HPV infection, cytological and histological features, and aneuploidy. In contrast, irrespective of the severity of the lesion, patients exhibiting the mutant *PDCD1* -606A allele at single or double doses exhibited increased protein and gene expression when compared to the *PDCD1* -606GG wild type genotype. Besides, the presence of HPV was associated with the decrease in *PDCD1* expression and PD-1 levels in carriers of the -606 A allele presenting severe lesions, suggesting that other mediators induced during the HPV infection progression may play an additional role. This study showed that increased PD-1 levels are influenced by the -606G>A nucleotide variation, particularly in low-grade lesions, in which the A allele favors increased *PDCD1* expression, contributing to HPV immune system evasion, and in the high-grade lesion, by decreasing tissue PD-1 levels.

## Introduction

Human papillomavirus (HPV) is the most common sexually transmitted biological agent, responsible for causing several types of cancer, particularly in the anogenital region, accounting for about 85% of the cervical tumors (World Health Organization, 2017; De Oliveira et al., 2019). The high-risk oncogenic HPVs have developed immune system evasion mechanisms, favoring viral persistence and chronic inflammation, which play an important role in the progression from cervical injury to cancer (Aggarwal et al., 2006; Grivennikov et al., 2010; Senba and Mori, 2012; Marinelli et al., 2019).

The programmed cell death protein (PD-1), together with its ligands PD-L1 or PD-L2, exerts an inhibitory effect on the cellular immune response, maintaining the balance between activation and tolerance of the immune cell function. The PD-1/PD-L1 signaling pathway has been used by microorganisms and tumor cells to decrease host immune system activity, permitting chronic infection, cell transformation into the tumor, and tumor cell survival (Ishida et al., 1992; Keir et al., 2008). PD-1 protein is mainly expressed on the membrane of T- and B- lymphocytes, NK cells, dendritic cells, activated monocytes, and immature Langerhans cells (Boussiotis, 2016).

Belonging to the immunoglobulin superfamily, PD-1 is a type I transmembrane monomeric protein, which has a cytoplasmic tyrosine-based inhibitory motif (ITIM) and a tyrosine-based switch motif (ITSM) that transmit inhibitory signals to the immune system cells. Once the peptide-MHC complex on the surface of the antigen-presenting cell (APC) binds to the T-cell receptor (TCR), the PD-L1 expressed on APC binds to the PD-1 receptor on T-cells and induces the phosphorylation of ITIM and ITSM motifs. The recruitment of the SHP-1 and SHP-2 phosphatases causes dephosphorylation of other signaling molecules of the cascade, inhibiting phosphatidylinositol 3-kinase (PI3K) and protein kinase B (Akt). These events culminate in immune response inhibition, reflected by i) decreased production of cytokines, such as IFN-γ, IL-2, and TNF-α, ii) inhibition of proliferation and survival of T cells, and iii) re-establishment of the immunological homeostasis, decreasing the expression of co-stimulatory molecules at the immunological synapsis (Muenst et al., 2016; Salmaninejad et al., 2018). Nevertheless, the PD-1/PD-L1 signaling pathway may be used by tumor cells to attenuate or escape anti-tumor immunity, facilitating tumor progression. In human malignancies, high T-cell PD-1 expression has been reported in Hodgkin’s lymphoma, chronic lymphocytic leukemia, and breast, bladder, and ovarian cancers, suggesting a state of functional exhaustion of T cells (Muenst et al., 2016; Hollander et al., 2018; Kawahara et al., 2018; Lewinsky et al., 2018; Wieser et al., 2018; Jiang et al., 2019).

The *PDCD1* gene is located at chromosome 2 (2q37.3), presents 5 exons, and encodes a 288 amino acid PD-1 protein. Alternative splicing can generate different isoforms that are expressed at similar levels after T cell activation (Shinohara et al., 1994; Keir et al., 2008); however, genetic variants at *PDCD1* coding and regulatory 5’ and 3’ untranslated regions (UTR) may influence protein levels and the natural history of cancer development (Tao et al., 2017; Wang et al., 2018). Several *PDCD1* polymorphic sites have been described, including i) 298 single nucleotide polymorphisms (SNPs) at the coding region, of which 213 missense, 112 synonymous, 7 nonsense, and 4 frameshift mutations; ii) 512 SNPs at the extended 5’UTR, iii) 490 SNPs at the extended 3’UTR, and iv) 1,791 intronic sequence mutations. Among these SNPs, 36 at the coding region, 57 at 5’UTR, 56 at 3’UTR and 283 intronic ones have clinical importance (https://www.ncbi.nlm.nih.gov/SNP/). Among all these polymorphic sites, the *PDCD1* promoter region -606G>A polymorphism (rs36084323) has been associated with the oncogenic p53 protein in breast cancer (Hua et al., 2011), in measles-induced autoimmune neurological manifestations (Ishizaki et al., 2010), and the susceptibility to hepatitis B infection (Hou et al., 2017).

To study the role of PD-1 on the progression of cervical lesions, we evaluated PD-1 tissue and *PDCD1* gene expression in women infected or not by HPV. To understand the contribution of genetic factors on PD-1 and *PDCD1* expression, we evaluated the *PDCD1* promoter region -606G>A polymorphism (rs36084323) in these patients.

## Materials and Methods

### Study Population and Ethical Consideration

The study population encompassed 295 women, aged 18-71 years (median=37 years). Among the 107 HPV-infected women, 90 were infected by high-risk, 11 by low-risk HPV, and in 6 samples the HPV viral genotype was not identified. Patients attending the Integrated Health Center Amaury de Medeiros (CISAM) and in the Professor Fernando Figueira Institute of Integral Medicine (IMIP), in Pernambuco, Brazil, between April 2016 to October 2018, were invited to participate in this study during the routine gynecological consultations for evaluation of the Papanicolaou smears, which are performed annually in asymptomatic and symptomatic women. This study was approved by the Ethics Committee of the Aggeu Magalhães Institute (CAAE: 51111115.9.0000.5190), and all participants signed an informed consent form after receiving a detailed explanation about the research. HIV-positive patients were not included in this study.

Clinical and laboratory data were obtained from medical records and interviews, using a standard questionnaire (**Table 1**). Venous blood and cervical exfoliative cells and biopsies were obtained during routine colposcopy analysis and evaluated by experienced gynecologists.

**Table 1:** Demographic, clinical, and laboratory features of women exhibiting cervical lesion associated with the HPV-infections [low-grade squamous intraepithelial lesion (LGSIL), the high-grade squamous intraepithelial lesion (HGSIL) and cervical intraepithelial neoplasia (CIN)] or non-associated with the HPV infection [atypical squamous cells of undifferentiated (ASC-US), atypical squamous cells not excluding high-grade squamous intraepithelial lesion (ASC-H)].

Patients characteristics	TOTAL		HPV +		HPV -		
	N = 295	%	N = 107	36.3%	N = 188	63.7%	
**Age - years**							
Median (minimum - maximum)	37.8 (18-70)		36 (18-66)		38 (19-72)		*P*= 0.3066
**Use of oral contraceptives**	293		107		188		
Yes	68	23.2	19	17.9	49	26.2	*P*= 0.1152
No	225	76.8	87	82.1	138	73.8	
Data missing	2		1		1		
**Cytological alterations**	255		107		188		
No atypias	104	40.8	24	25.5	80	49.6	*P<* 0.0001
ASC-US and ASC-H	26	10.2	6	6.4	20	12.4	
LGSIL	51	20.0	22	23.4	29	18.0	
HGSIL	74	29.0	42	44.7	32	19.9	
Data missing	40		13		27		
**Histological alterations**	267		107		188		
Uninjured (not submitted to biopsy)*	74	27.7	11	11.7	63	36.4	*P<* 0.0001
Benign injury	41	15.4	9	9.6	32	18.5	
CIN I	23	8.6	8	8.5	15	8.7	
CIN II	67	25.1	33	35.1	34	19.6	
CIN III	62	23.2	33	35.1	29	16.8	
Data missing	24		13		15		
**Cellular ploidy**	122		107		188		
Aneuploidy	27	22.1	10	23.8	63	78.8	*P<* 0.0001
Diploidy	95	77.9	32	76.2	17	21.2	
Data missing	173		65		108		

To evaluate possible differences between the groups of infected and uninfected by HPV, Mann-Whitney test (age-years), Chi-square test (cytological and histological alterations), and Fisher test (Use of oral contraceptives and Cellular ploidy) were performed. *According to the Brazilian Ministry of Health’s screening policy for cervical cancer, there is no indication for colposcopy for these patients, due to the absence of changes in the cytological examination.

For the *PDCD1* gene expression analysis, the reference group was women presenting no atypia in the cytopathological Papanicolaou smear, who were not eligible to be subjected to biopsy due to ethical restriction. Abnormal cytology was classified using the Bethesda system. Cervical abnormalities were stratified as the low-grade squamous intraepithelial lesion (LGSIL) and high-grade squamous intraepithelial lesion (HGSIL). Women presenting abnormal cytology were subjected to cervical biopsies for histological stratification into the benign lesions, low-grade cervical intraepithelial neoplasia (CIN) I, and high-grade CIN II and CIN III. For the immunohistochemistry evaluation of PD-1 protein levels, the reference controls were specimens from the uninjured area adjacent to the lesion.

### Histopathology

Biopsies of the cervical lesion and the adjacent area were fixed in formalin (10%) and embedded in paraffin. Four μm tissue sections were cut using a manual microtome (American Optical, Rotary, Leica, Buffalo Grove, IL), placed on silanized glass slides (Agilent-Dako, Santa Clara, CA), stained with hematoxylin-eosin (HE) and mounted with the medium Entellan^®^ (MERCK, Burlington, MA). The sections were visualized with 400x magnification in an inverted microscope (Zeiss, Göttingen, Germany) equipped with a camera and with a 4.7.4 Image Analysis Program (AxionCam MRm Zeiss). HE-stained slides were blindly evaluated by a cervical pathologist.

### HPV Detection and Typing

Genomic DNA was extracted from 500μL of a cervical cell suspension, using the Illustra Blood kit (Healthcare^®^, Little Chalfont, Buckinghamshire, UK), according to the manufacturer’s instructions, and quantified using the NanoDrop 2000 spectrophotometer (ThermoScientific, Waltham, MA). The quality of the extracted DNA was also assessed by PCR-amplification using the human constitutive glyceraldehyde phosphate dehydrogenase (*GAPDH*) gene (Martins et al., 2014). HPV infection in cervical samples was diagnosed by amplifying a fragment of the viral *L1* gene with the degenerate MY09 and MY11 primers (Manos et al., 1989), using the L1-fragment encoded plasmid as the positive control. A reaction without adding any sample was used as a negative control. The presence of a band of approximately 450 base pairs (bp) in the 2% agarose gel confirmed the presence of the viral infection. Each amplification product was directly sequenced with the MY11 primer in the Genetic AnalyzerABI 3500 (Applied Biosystems, Foster City, CA) sequencer, using the BigDye terminator v3.1 cycle sequencing kit (Applied Biosystems). The chromatograms were visualized in the Mega 6.0 program (Tamura et al., 2011) to assess the quality of the sequence. Samples with defined peaks and low background in the chromatogram were submitted to Papillomavirus Episteme (https://pave.niaid.nih.gov/) to HPV genotyping.

### Determination of Cellular Ploidy

Cervical cells of the uterine cervix (150μL) were ruptured using 2mL of Pharm Lyse lysis buffer (Becton Dickinson, Franklin Lakes, NJ), vigorously homogenized, and incubated in the dark for 10 minutes, and centrifuged for 120 seconds at 1,000 x g. The supernatant was discarded and the pellet resuspended in 2mL of FACs flow buffer, gently homogenized, and recentrifuged for 120 seconds at 1,000 x g. After discarding the supernatant, the pellet was resuspended in 500 μL of propidium iodide plus 10 μL of RNase (100 μg/mL), and incubated at room temperature for 30 minutes at 4°C and then for 10 minutes at 8°C. DNA fluorescence was measured by laser excitation at 488 nm and emission above 600 nm. The DNA index was estimated by comparing the proportion of DNA from the cervical cells analyzed with the diploid blood cells, using the software ModFitLT V3.0 (Verity Software House Inc., Topsham, ME). Aneuploidy was defined by a deviation in the DNA histogram in more than 10% of the cell population analyzed in the area corresponding to G0- G1 of the cell cycle in the sample (Martins et al., 2014).

### Cervical Cell PD-1 Levels

Immunohistochemistry (IHC) analysis for cervical biopsies was manually performed using the DAKO EnVision ™ FLEX kit (Agilent-DAKO, Santa Clara, CA). For antigenic recovery, tissue was pretreated with citrate buffer, pH 6.1 (Agilent-DAKO), and heated for 30 minutes. After blocking with endogenous peroxidase, the tissue sample was incubated with the primary monoclonal anti-PD-1 mouse antibody (ABCAM, Cambridge, UK) diluted (1:100) with DAKO antibody diluent for 1h. After washing, the sections were incubated with secondary antibody for 20 minutes and then visualized with the DAB reagent (3,3’-diaminobenzidine tetrahydrochloride, DAKO). After labeling, tissue sections were counterstained with Harris’ hematoxylin and assembled with Entellan^®^ (MERCK).

The IHC slides of the cervical lesion and the adjacent uninjured area were independently analyzed by two specialist pathologists. Cell areas showing brown staining were considered to be positive for the expression of PD-1 and quantified in three fields showing the highest labeling per slide, using the Gimp 2.10.18 software (GNU Image Manipulation Program, UNIX platforms, www.gimp.org). To minimize possible reading errors, we measured pixels in areas with an intense and less intense stained area in the same picture; and combined both readings to generate the final PD-1 expression value.

### Cervical Cell *PDCD1* Gene Expression

Total RNA was extracted from a 1000 μL of cervical cell suspension, using Trizol^®^ reagent (Invitrogen), and submitted to cDNA synthesis using the MLLV reverse transcriptase (Invitrogen), accordingly to the manufacturer’s instructions. For *PDCD1* expression, the qPCR was prepared with 1μL cDNA and 10 pmoles of each PD1F: 5’ GAT GGT TCT TAG ACT CCC CAG ACA G 3’, and PD1R: 5’ GGC TCA TGC GGT ACC AGT TTA GCA C 3’ primers, in Power SYBR™ Green PCR Master Mix (Applied Biosystems, Foster City, CA). For the expression of the *GAPDH* constitutive gene, we used also 1 μL cDNA and 10 pmoles of GAPDH2F: 5’ AGA AGG CTG GGG CTC ATT TG 3’ and GAPDH2R: 5’ GTG GTC ATG AGT CCT TCC AC 3’ primers in Power SYBR™ Green PCR Master Mix. All primers were designed nearby the exon-intron junction to amplify a fragment that covers two exons, assuring amplification of the cDNA target. The qPCR was performed in a final volume of 20 μL containing 10 μL of 2× Power SYBR^®^ Green PCR Master Mix, 1 µl forward and 1 µl reverse PCR primers (500nM), 1μL cDNA and 7 µL nuclease-free water. The reaction mixtures were processed with an initial holding period at 95°C for 10 min, followed by a two-step PCR program for 40 cycles that consisted of 95°C for 15 sec and 60°C for 1 min. The *PDCD1* and *GAPDH* calibration curves showed similar amplification efficiency, and samples were evaluated in duplicate in Quant Studio 5 (Applied Biosystem). Only samples showing a melting curve with single and specific peaks, and only duplicates showing standard deviation less than 0.5 were considered for analyses. A unique threshold was settled for each gene amplification in all plates, and the sample CTs were annotated. *PDCD1* relative expression was determined by ΔCT-comparative quantification, in which *PDCD1* expression was normalized by the endogenous gene expression (ΔCT =CT_PDCD1_ – CT_GAPDH_) for each sample, and the final results were expressed in fold-change, using the equation (Fold-change=2^-ΔCT^).

### *PDCD1* Promoter Region Polymorphism

DNA from peripheral blood mononuclear cells, extracted using DNAzol^®^ Reagent (Invitrogen, Carlsbad, CA) was used for the detection of the -606G>A *(*rs36084323) SNP. Briefly, DNA was amplified using the *PD-1* PROMO F (5 ‘GAA AGA TCT GGA ACT GTG GC 3’) and PD-1 PROMO R (5 ‘TGA GAG TGA AAG GTC CCT CC 3’) primers. The amplification reaction was performed in a final volume of 20 μL containing 1x of polymerase buffer (Applied Biosystems), 0.5 mM MgCl_2_, 2% DMSO, 200 μM dNTP’s, 1.0 μM of each primer, 1.0 unit of Ampli-Taq Gold (Applied Biosystems) and 80-200 ng of genomic DNA for the amplification of a 962 bp-PD1 fragment. The cycling conditions included an initial stage at 94°C for 10 min; 40 cycles of denaturation at 94°C for 1 min, annealing at 62 °C for 1 min and extension at 72 °C for 1.2 min, and final extension for 7 min at 72°C. The PCR product was visualized using a 1.5% agarose gel and, subsequently, sequenced by the SANGER method, following the BigDye protocol on ABI 3500 sequencer (Applied Biosystems). Polymorphic sites were determined using the Seqman^®^ program (Roche 454, Life ScienceTM, Branford, CT) and individually annotated in an Excel 2016 spreadsheet.

### Bioinformatics Analysis

To propose a list of possible microRNAs (miRNA) associated with the *PDCD1* rs36084323 SNP, we took advantage of two separated approaches using: i) the mirDIP Version 4.1.11.1 (Tokar et al., 2018), which integrates 30 different databases of miRNA target prediction, together with a unidirectional search query with *PDCD1* (PD-1 alias), to search for all predicted miRNAs without filtering any specific ‘Score class’; ii) the sequence of 100 base pairs that surrounds the SNP, as retrieved from Genome Browser Gateway (http://genome.ucsc.edu/), and blasted using miRBase Release 22.1 (Kozomara et al., 2019). Then, we selected the miRNAs that annealed with the SNP site taking into account the two possible alleles.

### Statistical Analysis

Association analyses of allele and genotype frequencies with clinical variables were performed using the two-tailed Fisher’s exact and chi-square tests, considering a significance level of *P <*0.05. The Hardy-Weinberg Equilibrium was assessed by the Online Encyclopedia for Genetic Epidemiology (OEGE). The D’Agostino-Pearson test was used to assess the homogeneity of the PD-1 expression in pixels and fold-change. The central tendency was expressed as a median and the Kruskal-Wallis and Mann-Whitney tests were used to compare numeric variables. The graphics were prepared using GraphPad Prism Software version 5.0 for windows (www.graphpad.com, La Jolla, CA).

## Results

### PD-1 Detection in Cervical Samples

**Figure 1:**
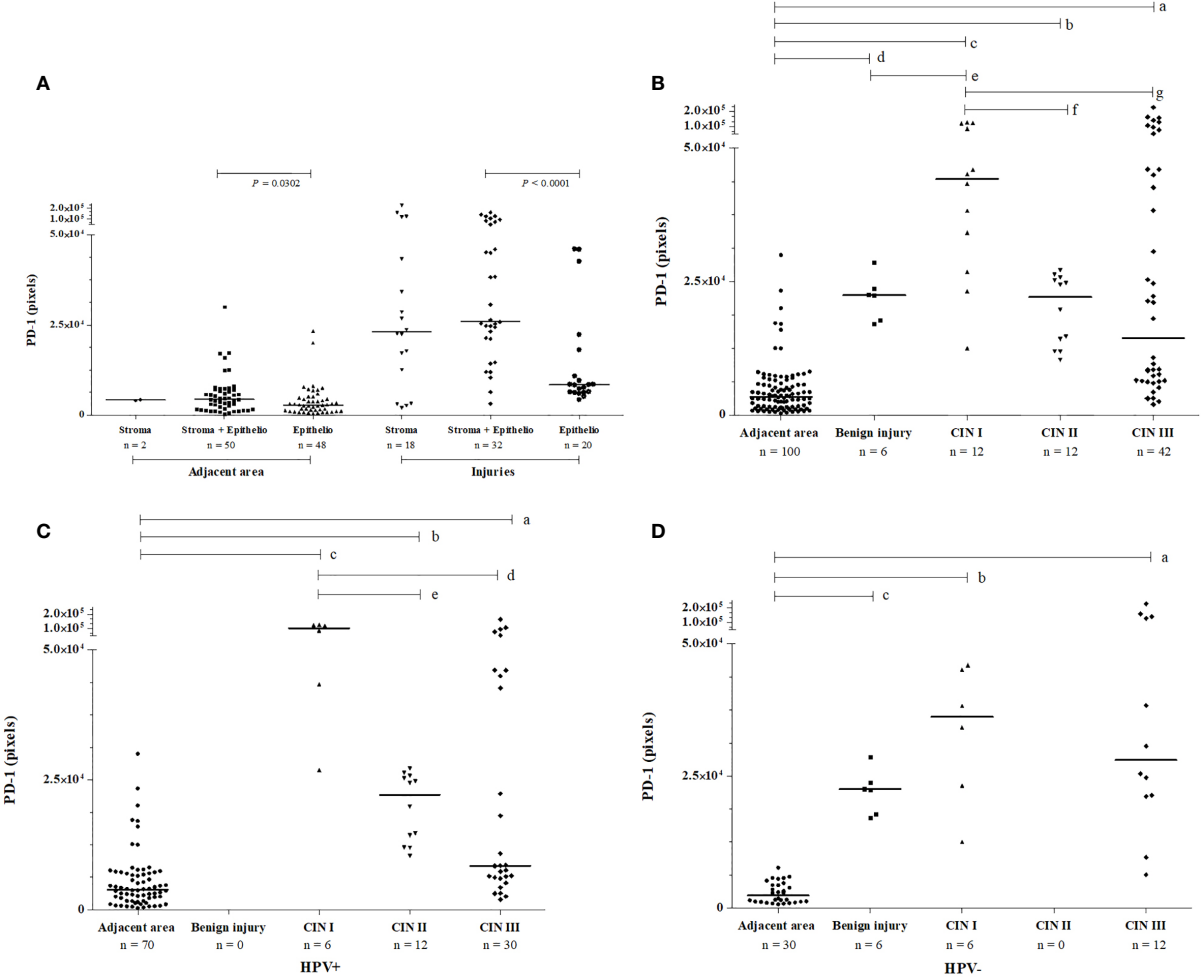
Pattern of PD-1 immunochemistry labeling observed in cervical samples, according to **(A)** the tissue location in injured and uninjured adjacent areas, **(B)** the severity of lesion irrespective to HPV infection, **(C)** in the presence of HPV infection, and **(D)** the absence of HPV infection. PD-1 labeling was analyzed by two independent pathologists, and the intensity of the labeling (pixels) was quantified in three fields per slide. Data were presented as medians, and the comparisons were performed using the Kruskal-Wallis test followed by Mann Whitney U test. In graph B, PD-1 levels were high irrespective of the severity of cervical lesions, CIN III (in a, *P*<0.0001); CIN II (in b, *P*<0.0001); CIN I (in c, *P*<0.0001); and benign lesions (in d, *P*<0.0001) compared to the uninjured adjacent area; and between benign lesion vs. CIN I (in e, *P* = 0.0131); however, CIN I lesions showed the highest PD-1 level compared to the CIN II (in f, *P* = 0.0014) and CIN III (in g, *P* = 0.0172). In graph C, in presence of HPV the PD-1 levels were high in CIN III (in a, *P*<0.0001), CIN II (in b, *P*<0.0001), and CIN I (in c, *P*<0.0001) compared to uninjured tissue, and lower PD-1 levels were observed in CIN III (in d, *P* = 0.0048) and CIN II (in e, *P* = 0.0012) compared to the levels in low-grade lesion CIN I. In graph D, in women non-infected by HPV, high PD-1 levels were also observed in CIN III (in a, *P*<0.0001), CIN I (in b, *P* = 0.0001), and benign injury (in c *P* = 0.0001) compared to the uninjured adjacent area.

PD-1 expression was evaluated in samples exhibiting HPV infection and in samples without HPV infection. Irrespective of lesion severity, we observed three patterns of PD-1 staining in cervical samples: i) exclusive labeling of the stratified squamous epithelium, ii) exclusive labeling of stromal cells, and iii) labeling of epithelium and stroma. Considering the uninjured adjacent areas, the expression of PD-1 in epithelium and stroma (n = 50, median = 4,313 pixels) was higher when compared to sections that labeled only the epithelium (n = 48, median = 2,810 pixels, *P* = 0.0302). Considering the injured areas (cervical lesions), the expression of PD-1 in epithelium and stroma (n = 32, median = 21,184 pixels) was also significantly higher when compared to specimens that exclusively labeled the epithelium area (n = 20, median = 6,339 pixels, *P* < 0.0001) (**Figure 1A**).

**Figure 2:**
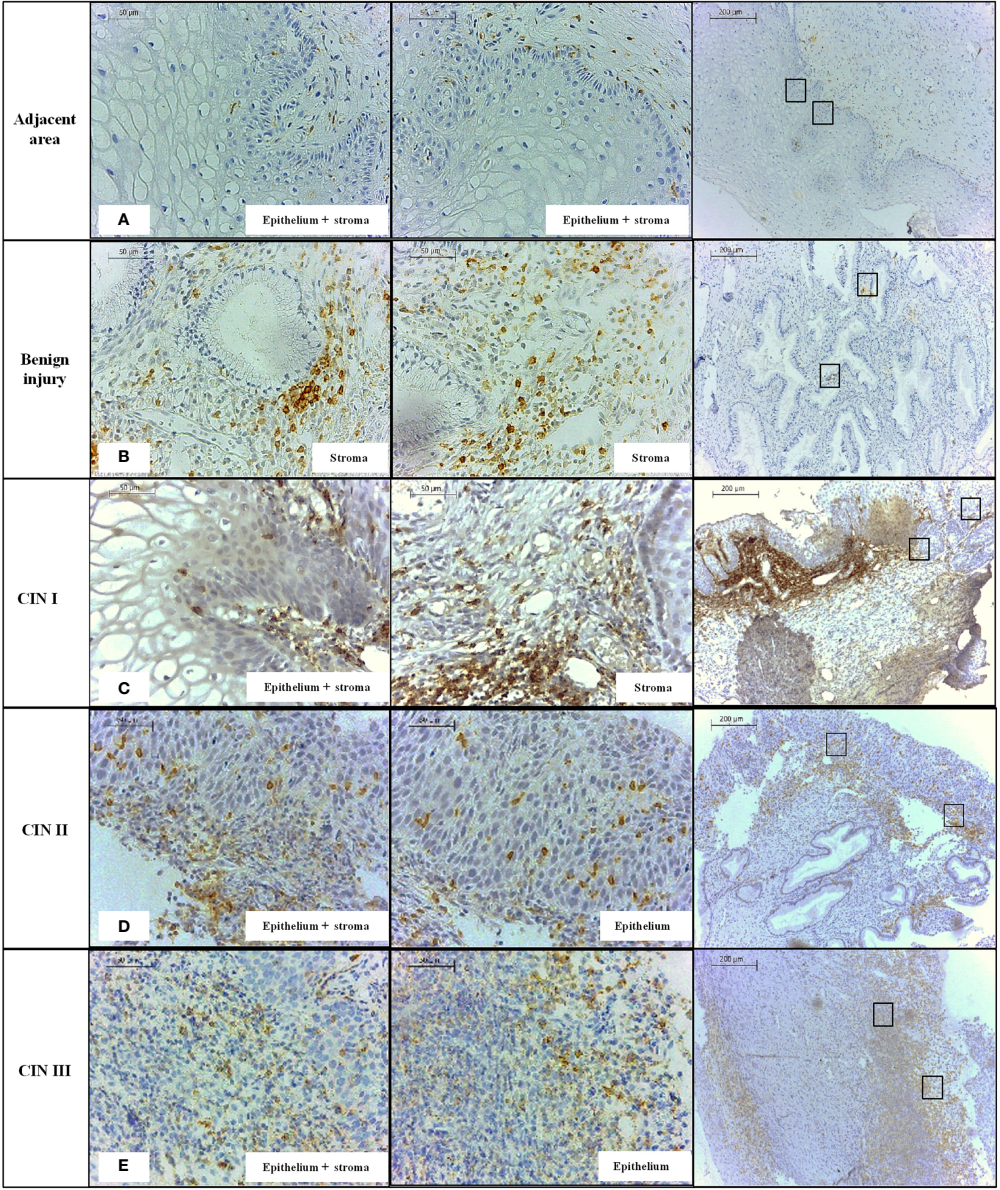
Immunohistochemistry labeling of PD-1 level observed in specimens obtained from the mucosa of the uterine cervix, stratified according to the presence or not of the lesion, and according to the severity of the cervical lesion. **(A)** cervix without lesion, **(B)** benign injury, **(C)** CIN I, **(D)** CIN II, **(E)** CIN III, at 100X and 400X magnifications. PD-1 protein was primarily detected in the stratified epithelium and stroma of the cervical mucosa.

The evaluation of PD-1 protein level according to the severity of the lesion revealed the following results: i) PD-1 expression in benign lesions, and cervical intraepithelial neoplasia (CIN I, CIN II, and CIN III) was significantly higher when compared to uninjured adjacent tissue (*P*<0.0001, for each comparison, **Figure 1B**); ii) the PD-1 expression in benign lesions did not differ from CIN II (*P* = 0.7431) and CIN III (*P* = 0.5854) and, similarly, CIN II did not differ from CIN III (*P* = 0.5122); and iii) the PD-1 levels in low-grade lesions (CIN I) (n = 12, median = 44,215 pixels) were higher than in those presenting high-grade (CIN II and CIN III) lesions (n = 54, median = 18,942 pixels, *P* = 0.0046); however, the pattern of expression was different. PD-1 labeling in low-grade lesions was higher in infiltrating immune cells of the stroma compared to the epithelium, whereas in high-grade lesions PD-1 expression was observed primarily in the epithelium (**Figure 2**). Additionally, irrespective of the severity of the lesion, PD-1 levels were increased compared to adjacent uninjured areas. Also, in CIN I the presence of HPV was associated with increased PD-1 protein levels (*P* = 0.0649), whereas in CIN III was associated with decreased levels (*P* = 0.0148) compared to correspondent tissue lesion in absence of HPV infection (**Figures 1C, D**).

### Polymorphism of the *PDCD1* Promoter Region

Irrespective of the severity of the cervical lesion, the frequency of the wild -606G allele was 91.3%, and the distribution of the GG (83.7%), GA (15.2%), and AA (1.1%) genotypes adhered to the Hardy-Weinberg equilibrium (χ² = 0.48). Taking into account the low frequency of the AA genotype, we lumped together the AA and GA genotypes to allow statistical analysis. Women carrying the -606A allele were not at increased risk for i) development of HPV infection, ii) exhibiting cytological and histological HPV and non-HPV changes, and ii) presenting aneuploidy (**Table 2**).

**Table 2:** Allelic and genotypic frequency of the promoter region *PDCD1*-606 G>A (rs36084323) polymorphism observed in women exhibiting grades of cervical lesions, stratified according to i) presence or not of the HPV infection, ii) cytological/histological alterations, and iii) cellular ploidy.

Patients characteristics	*PDCD1* (rs36084323)											
	GG		GA+AA		*P*	OR (CI-95%)	G		A		*P*	OR (CI-95%)
	N= 231	83.7%	N=45	16.3%			N= 504	91.3%	N=48	8.7%		
**HPV Infection**												
Yes	83	35.9	17	37.8	0.8659	0.92 (0.48-1.79)	182	36.1	18	37.5	0.8757	0.94 (0.51-1.74)
No	148	64.1	28	62.2			322	63.9	30	62.5		
Total	231	100.0	45	100.0			504	100.0	48	100.0		
**Cytological alterations**												
HGSIL	55	27.8	16	40.0	0.3018	n/a	125	28.8	17	40.5	0.2864	n/a
ASC-US, ASC-H and LGSIL	62	31.3	10	25.0			133	30.6	11	26.2		
No atypias	81	40.9	14	35.0			176	40.6	14	33.3		
Total	198	100.0	40	100.0			434	100.0	42	100.0		
**Presence of histological alterations**												
Presence of CIN	118	56.5	23	56.1	1.0000	1.01 (0.52-1.99)	259	56.8	23	52.3	0.6337	1.20 (0.64-2.23)
Absence of CIN	91	43.5	18	43.9			197	43.2	21	47.7		
Total	209	100.0	41	100.0			456	100.0	44	100.0		
**Histological alterations**												
CIN III	48	23.0	10	24.4	0.8669	n/a	106	23.2	10	22.7	0.7133	n/a
CIN II	51	24.4	11	26.8			113	24.8	11	25.0		
CIN I	19	9.1	2	4.9			40	8.8	2	4.5		
Benign injury	33	15.8	8	19.5			72	15.8	10	22.7		
Uninjured	58	27.8	10	24.4			125	27.4	11	25.0		
Total	209	100.0	41	100.0			456	100.0	44	100.0		
**Cellular ploidy**												
Aneuploidy	22	24.2	3	12.0	0.2737	2.34 (0.64-8.57)	47	22.8	3	11.5	0.3092	2.27 (0.65-7.88)
Diploidy	69	75.8	22	88.0			159	77.2	23	88.5		
Total	91	100.0	25	100.0			206	100.0	26	100.0		

Atypical squamous cells of undetermined significance (ASC-US), atypical squamous cells-not excluding high-grade squamous intraepithelial lesion (ASC-H), the low-grade squamous intraepithelial lesion (LGSIL), the high-grade squamous intraepithelial lesion (HGSIL), cervical intraepithelial neoplasia (CIN). N, sample number; P, P-value; G, wild allele; A, variant allele; OR, odds ratio and 95%CI, confidence interval. The frequencies of alleles and genotypes were compared using the two-tailed Fisher’s exact and Chi-square tests.

Considering the differential exposure of HPV according to age and the risk for developing cervical lesions, we evaluated the influence of the patient age on the studied variables. Age did not influence the *PDCD1* genotype frequency among patients presenting or not HPV (*P* = 0.3066), and among patients exhibiting benign injury (*P* = 0.1692), CIN I (*P* = 0.5698), CIN II (*P* = 0.0746) or CIN III (*P* = 0.7366) compared to women with normal cytomorphological results.

### Association of the *PDCD1* Gene Expression in the Cervical Lesion With *PDCD1* Polymorphism

**Figure 3:**
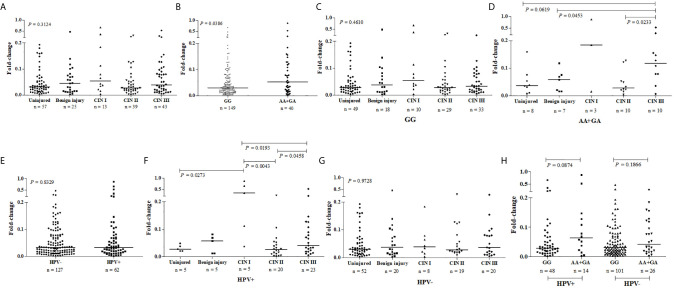
*PDCD1* mRNA expression in exfoliative cervical cells of patients presenting normal (uninjured) and abnormal cytomorphological smear (injured) stratified according to the severity of the lesion, presence or absence of HPV infection, and the nucleotide variability at the promoter region *PDCD1*-606G>A polymorphic site (rs36084323). *PDCD1* expression was calculated by the qPCR comparative method using as reference the *GAPDH* gene and the SYRB green detection system, performed in duplicate. Fold-change differences were estimated by the Kruskal-Wallis or Mann-Whitney U tests. **(A)** *PDCD1* expression stratified according to the severity of the cervical lesion, **(B)** *PDCD1* expression stratified according to the presence of the -606GG and AA/GA genotypes, **(C)** *PDCD1* expression among carries of -606GG genotype, **(D)** and carries of -606 A allele in homo or heterozygosis stratified according to the severity of the cervical lesion, **(E)** *PDCD1* expression among women infected and non-infected by HPV, and stratified according to the severity of the cervical lesion in the presence **(F)** or absence **(G)** of HPV infection, and **(H)** *PDCD1* expression stratified according to the presence of the -606GG and AA/GA genotypes in the presence or absence of HPV infection.

The presence of cervical injury was not associated with a greater *PDCD1* expression by exfoliative cervical cells when compared to cervical samples from women with a normal cytomorphological smear (**Figure 3A**). However, considering samples altogether, the mutant -606A allele in single or double dose was associated with higher *PDCD1* gene expression in cervical cells compared to the wild type -606GG genotype (**Figure 3B**). There was no statistical difference (*P* = 0.4692) between *PDCD1* expression levels in lesions of different severities in carriers of the -606GG genotype (**Figure 3C**). However, among carriers of the -606A allele in homo or heterozygosis, the expression of *PDCD1* was higher in exfoliative cells of patients exhibiting high-grade CIN III lesions compared to those presenting benign (*P* = 0.0453) or CIN II (*P* = 0.0233) lesions (**Figure 3D**). Moreover, despite no association of HPV-infection with *PDCD1* expression (P=0.8329) was observed (**Figure 3E**), the presence of HPV increased *PDCD1* expression, particularly in women presenting CIN I lesion (**Figures 3F, G**), a finding that may have been influenced by the -606A allele (**Figure 3H**).

### Association Between PD-1 Protein Levels and *PDCD1* Polymorphism

**Figure 4:**
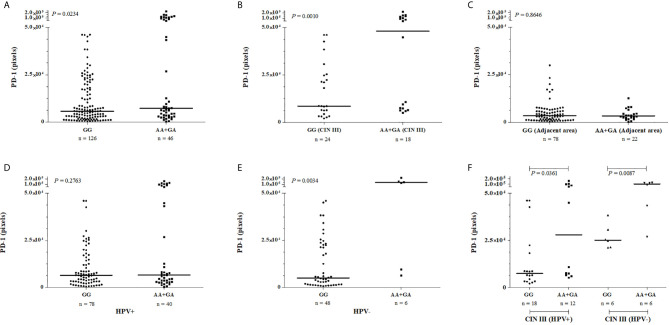
PD-1 levels in cervical mucosa of samples obtained from patients exhibiting cervical lesions, stratified according to the severity of the lesion, presence or absence of HPV infection and according to the *PDCD1* -606G>A (rs36084323) polymorphism. The immunohistochemistry data were analyzed by two pathologists independently, and the intensity of labeling was quantified in three fields per slide. Data were presented as medians. The median differences were calculated using the Mann-Whitney test. **(A)** PD-1 protein levels were increased cervical mucosa of women encompassing the -606AA and -606GA genotypes when compared to the -606GG genotype, the difference is high among severe CIN III injury **(B)**, but not in the uninjured adjacent tissue **(C)**. In the presence of HPV infection **(D)**, the high PD-1 levels previously associated with the presence of the -606A allele **(E)** was not observed anymore; however, the influence of HPV infection on PD-1 levels continue to be observed for high-grade CIN III lesions even being in less magnitude **(F)**.

We also evaluated whether the PD-1 tissue levels were associated with the presence of the rare *PDCD1* -606G>A polymorphic sites. Considering the dominant model for the rare allele, women carrying the A allele in homozygosis or heterozygosis (n = 46, median = 7,381 pixels) exhibited increased PD-1 levels in cervical samples when compared to those homozygous for the *G* allele (n = 126, median = 5,697 pixels, *P* = 0.0234) (**Figure 4A**). Besides, the association of *PDCD1* -606A allele with the PD-1 expression was strengthened when we specifically evaluated women with high-grade cervical lesions (CIN III); i.e., women carrying the -606A allele at homo- or heterozygosis exhibited higher PD-1 levels in the cervical lesions when compared to women carrying the homozygous -606G allele (GA + AA with the median of 48,117 pixels *vs*. GG with the median of 8,539 pixels, *P* = 0.0010) (**Figure 4B**). However, PD-1 levels in uninjured adjacent tissue were not associated with the -606G>A variation site (GA + AA with the median of 3,358 pixels *vs*. *GG* with the median of 3,409 pixels, *P* = 0.8646) (**Figure 4C**). In the presence of HPV infection, the high PD-1 levels previously associated with the presence of the -606A allele was not observed anymore (**Figures 4D, E**); however, the influence of HPV infection on PD-1 levels continue to be observed for high-grade CIN III lesions even being in less magnitude (**Figure 4F**).

### Predicted miRNA and *PDCD1* rs36084323 SNP Association

**Figure 5:**
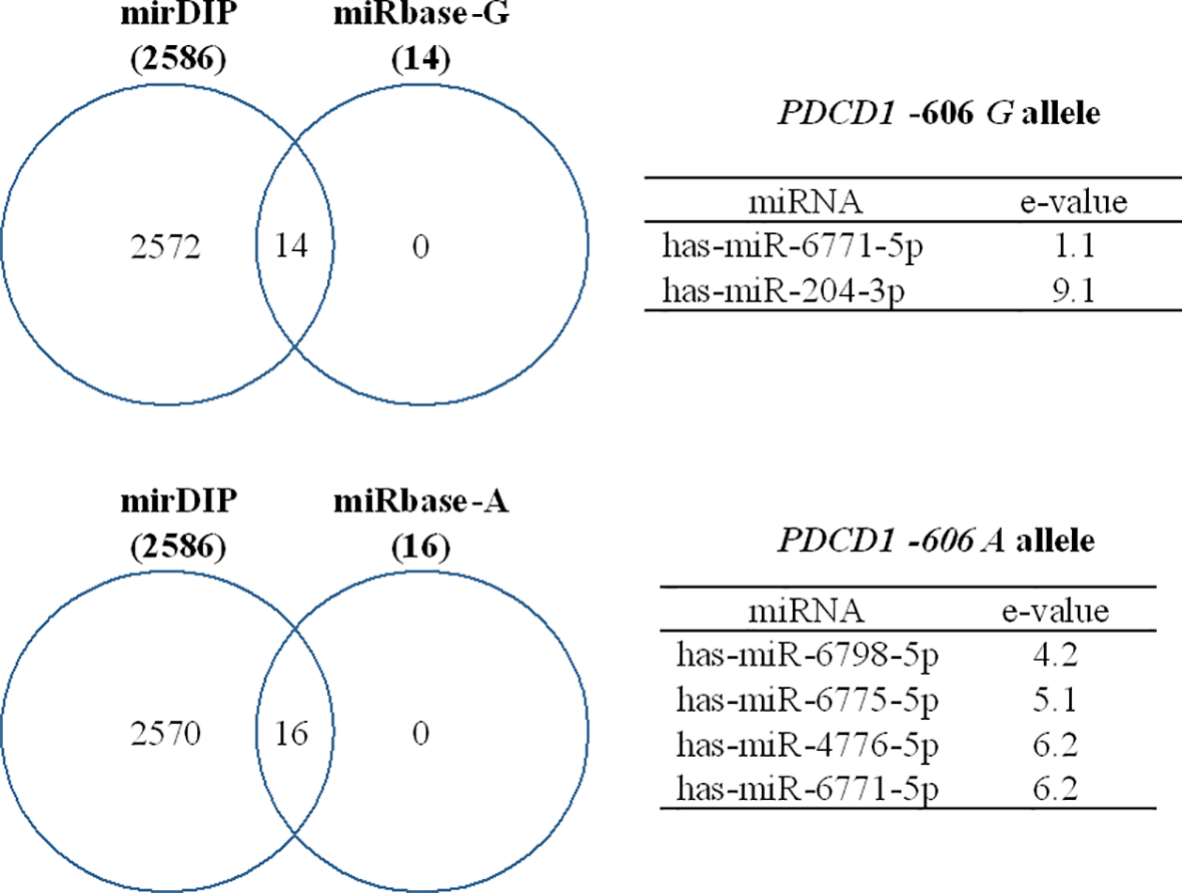
Predicted miRNAs that target the *PDCD1* -606G>A (rs36084323) polymorphic site. Binding affinity was evaluated using miRbase and mirDIP software and expressed as e-values, in which high affinity is represented by low e-values and vice-versa.

Considering the discrepancy regarding the results of the protein (**Figure 4F**) and gene expression (**Figure 3H**) levels, we further evaluated the differential targeting of miRNAs at the *PDCD1* -606 variation site. An in-silico study showed that the hsa-miR-204-3p binds exclusively to the *G* allele, whereas the hsa-miR-6798-5p, hsa-miR-6775-5p, and hsa-miR-4776-5p bind only to the A allele, and the hsa-miR-6771-5p targeted both alleles. Notably, the miRNAs that targeted the -606G>A variation site were included among the 2,586 miRNAs that have been predicted to target the *PDCD1* gene, according to the mirDIP analysis (**Figure 5**).

In summary, our results showed that the -606A allele is rare in our population and, considering the dominant model, women carrying the -606 AX genotype (X= A or G) are more likely to respond to HPV-induced cervical lesions with decreased production of PD-1, and the intensity and pattern of labeling is related to the degree of the cervical injury.

## Discussion

Since healthy cervical specimens are not easily available due to ethical reasons, in this study we evaluated PD-1 expression in cervical biopsies obtained from patients presenting several stages of the cervical lesion (injured areas) and used, as controls, the adjacent uninjured cervical areas. Irrespective of the severity of the lesion, injured cervical specimens overexpressed PD-1 when compared to the adjacent uninjured area, and particularly observed in the stromal layer (**Figure 1A**). Notably, samples presenting high-grade (CIN II-III) lesions expressed less PD-1 when compared to low-grade (CIN I) lesions (**Figure 1B**). PD-1 expression predominated in the stroma in low-grade lesions, and the epithelium in high-grade lesions (**Figure 2**). The stroma-rich T cell-infiltrate in low-grade cervical lesions associated with the increased tissue PD-1 (Hemmat and Bannazadeh Baghi, 2019) primarily reflect the severity of inflammation associated with HPV infection (Yang et al., 2013), whereas the expression at the epithelium strongly indicates the role of PD-1 on the progression of the cervical lesions (Yang et al., 2017; Chang et al., 2018; Medeiros et al., 2018).

Considering that the magnitude of the *PDCD1* gene expression has been associated with the *PDCD1* -606 G>A polymorphic site (Ishizaki et al., 2010), we further investigated the relationship between alleles/genotypes with the magnitude of PD-1 cervical expression. First, we observed that the *PDCD1* -606AA genotype was rare in our population (1.1%), and according to the data reported at the 1000 Genomes Phase 3 database (The 1000 Genomes Project Consortium, 2015), the promoter region *PDCD1* -606G>A (rs36084323) polymorphic site presents diverse allele frequency distribution in worldwide populations (1000 Genomes Project Phase 3 Allele Frequencies rs36084323 Snp; Auton et al., 2015), being frequent in Asians (1000 Genomes Project Phase 3 Allele Frequencies rs36084323 Snp), particularly in Chinese (21.9%) and Japanese (25%) populations (Hua et al., 2011; Sasaki et al., 2014; Hou et al., 2017). The presence of the -606A allele was not associated with the risk for the development of HPV infection, probably because of the low frequency of the mutant allele in our population. According to the frequency of the -606A in our population, a cohort of 1,834 individuals would be necessary to discriminate susceptibility/protection alleles.

Women carrying the -606A allele in homozygosis or heterozygosis showed a significant increase in the *PDCD1* gene expression and PD-1 protein level when compared to those who carry the -606G allele in homozygosis. In HPV-non-infected women, the *PDCD1* expression was irrespective of the severity of the lesion, but in the presence of HPV infection, the *PDCD1* expression was significantly increased in CIN I lesions, which may suggest a viral attempt to evade immune response against infection, favoring viral persistence (Yang et al., 2013). The PD-1 protein level in CIN I lesions was also high in HPV-infected women compared to non-infected women (*P*=0.0649). In contrast, HPV decreased the PD-1 protein levels in CIN III (*P*=0.0148), even though the protein levels remained significantly increased compared to the uninjured adjacent area, indicating that the HPV infection is associated with the cervical transformation (Martins et al., 2014). Noteworthy, the -606G>A variation site did not influence cervical PD-1 protein levels in adjacent uninjured tissue indicating that, besides the genetic background, local microenvironment factors such as local inflammation may have a role

The functional implication of the *PDCD1* -606G>A polymorphism on the magnitude of PD-1 production has been attributed to the target site of the ubiquitin-converting enzyme 2 (UCE-2) transcription regulator (GGCCG in position -610 to -606). The -606G allele was associated with higher relative expression of *PDCD1* mRNA in peripheral blood mononuclear cells of Japanese and Filipino patients exhibiting subacute sclerosing panencephalitis due to measles infection (Ishizaki et al., 2010), and with lower survival in patients with non-small cell lung cancer (Sasaki et al., 2014). Additionally, the -606GG genotype was associated with protection against the development and progression of breast cancer in women from Northeastern China; however, the A allele and the AA genotype were more frequent in patients exhibiting the p53 protein, a marker of biologically aggressive breast cancer (Hua et al., 2011). The *PDCD1* -606AA genotype was also associated with chronic hepatitis B virus (HBV) infection in the Chinese population, a viral infection that may progress to hepatocarcinoma (Hou et al., 2017). Altogether, these studies indicate a differential role of the *PDCD1* -606G>A polymorphic site according to the major subjacent cancer or viral disorder. In our study, we reported that the *PDCD1* expression and PD-1 protein levels were associated with the *-*606 A allele, and were modulated by HPV in severe tissue damage, suggesting that other mediators induced during the HPV infection progression may play an additional role.

Among the myriad of transcriptional and post-transcriptional elements that may differentially target gene polymorphic sites, the UCE-2 transcription regulator, which modulates the *PDCD1* -606 G>A polymorphic site (Ishizaki et al., 2010), has not been previously evaluated in the context of the progression of the HPV infection. On the other hand, several miRNAs have been associated with HPV lesion progression (Pardini et al., 2018; Pulati et al., 2019), and little is known regarding miRNA targeting the *PDCD1*-606 G>A region. Considering that virus infection may change the miRNA cell repertoire expression (Park et al., 2017; Chirayil et al., 2018; Del Mar Díaz-González et al., 2019), and that miRNA may also regulate gene expression at the transcriptional level (Lytle et al., 2007; Ørom et al., 2008; Place et al., 2008; Zhang et al., 2014), we conducted a bioinformatics analysis to predict miRNA interaction with the *PDCD1* -606G>A polymorphic site. Three miRNAs were predicted to bind to the mutant A allele: i) the hsa-MiR-6798-5p up-regulates decidual NK cells in recurrent spontaneous abortion (Sfera et al., 2015), ii) the hsa-MiR-6775 is reported to silence the transcription of the alpha 7-cholinergic nicotinic receptor gene (*CHRNA7)* expressed on lymphocyte surface and associated with lymphocyte anergy, T regulatory cell differentiation and immunologic tolerance, which consequently may predispose to cancer development (Li et al., 2018), and iii) the hsa-MiR-4776-5p specifically targets the nuclear factor Kappa B inhibitor beta (NFKBIB) mRNA in Influenza A virus-infected cells, leading to activation of NF-kB, and survival of infected cells (Othumpangat et al., 2017). The hsa-MiR-6771-5p binds to the -606 G alleles with high affinity (e-value = 1.1) and weakly to the mutant *A* allele (e-value = 6.2), and it is involved in ZIKA-associated microcephaly. This miRNA shares the same sequence of the ZIKV genome and human genes associated with microcephaly (McLean et al., 2017). Finally, the hsa-MiR-204-3p targets the wild *PDCD1* -606G allele, and several studies report that this miRNA is a protective factor against cancer development by different cellular mechanisms depending upon the cell origin (Cui et al., 2014; Koga et al., 2018; Li et al., 2019; Xi et al., 2020). Besides targeting the *PDCD1* gene, these miRNAs may also be associated with cell transformation induced by viruses and may share sequences associated with virus infection complications. Therefore, a balance between transcriptional and post-transcription factors together with genetic variability may account for the final result that may halt or permit virus spread and cell transformation.

Concluding, this study showed that PD-1 protein levels are increased in HPV-induced cervical lesions, irrespective of the severity of the injury. In CIN I lesions, the highest PD-1 levels were observed in the inflammatory infiltrating cells of the stroma, whereas in high-grade CIN III lesions, the high PD-1 expression was observed in epithelial cells. In CIN I, the high levels of PD-1 were associated with increased *PDCD1* expression in HPV-infected samples, whereas in CIN III the presence of HPV induced a decrease in *PDCD1* expression and of PD-1 levels in carriers of the -606A allele, suggesting the possible gene regulation by miRNAs. Indeed, we identified some miRNAs specifically targeting the -606A allele region, which may be modulated by the presence of HPV, and may be involved in the progression of the cervical lesion. Future studies are needed to validate the role of these miRNAs in cervical cancer pathogenesis.

## Data Availability Statement

The original contributions presented in the study are publicly available in NCBI using accession number PRJNA734653.

## Ethics Statement

The studies involving human participants were reviewed and approved by Ethics Committee of the Aggeu Magalhães Institute (CAAE: 51111115.9.0000.5190). The patients/participants provided their written informed consent to participate in this study.

## Author Contributions

MS, FM, NL-S, and ED conceived, designed the study, did the formal analysis, and wrote the paper. MS, NS, FG, TG, CP, and MCS conducted the experimental work. LP, MR, MM, and SW followed-up patients and performed cytopathological and coloscopy evaluations. NL-S and ED applied for financial support and managed the project. All authors contributed to the article and approved the submitted version.

## Funding

This work was supported by grants from i) Brazilian Health Ministry Project DECIT-FINEP, (Grants #1299-13; 401700/2015-1); ii) CAPES (PROCAD grant #88881-068436/2014-09 and Finance code 001); iii) Foundation for Science and Technology of the State of Pernambuco (FACEPE) (Grant #PROEP-APQ16804.01/15 and fellowship #IBPG-0849-4.01/16 to FM); iv) Brazilian National Council for Scientific and Technological Development (CNPq) (grants #310364/2015-9 and #310892/2019-8 to NL-S and #302060/2019.7 to ED). The funders had no role in study design, data collection, and analysis, decision to publish, or preparation of the manuscript.

## Conflict of Interest

The authors declare that the research was conducted in the absence of any commercial or financial relationships that could be construed as a potential conflict of interest.

## Abbreviations

Akt, Protein kinase B; ASC – H, Atypical squamous cells-cannot exclude high-grade squamous intraepithelial lesion; ASC-US, Atypical squamous cells of undetermined significance; CI, Confidence interval; CIN, Cervical intraepithelial neoplasia (I-III); CISAM, Integrated Health Center Amaury de Medeiros; DNA, Deoxyribonucleic acid; DMSO, Dimethyl sulfoxide; GAPDH, Glyceraldehyde phosphate dehydrogenase; HE, Hematoxylin-eosin; HBV, Hepatitis B virus; HIV, Human immunodeficiency virus; *HLA-G*, Human leukocyte antigen G; HPV, Human papillomavirus; HSIL, High-grade squamous intraepithelial lesion; IMIP, Professor Fernando Figueira Institute of Integral Medicine; IHC, Immunohistochemistry; ITIM, Immunoreceptor tyrosine-based inhibitory motif; ITSM, Immunoreceptor tyrosine-based switch motif; LGSIL, Low-grade squamous intraepithelial lesion; NSCLC, Non-small cell lung cancer; OEGE, Online Encyclopedia for Genetic Epidemiology; OR, Odds ratio; *P*, *P-*value; PBMC, Peripheral blood mononucleated cells; PCR, Polymerase chain reaction; PI3K, Phosphatidylinositol 3-kinase; PD-1/*PDCD1*, Programmed cell death 1; PD-L1/PD-L2, Programmed cell death ligand 1 or 2; SNP, Single nucleotide polymorphisms; SSPE, Subacute sclerosing panencephalitis; TAE, Tris-acetate-EDTA buffer; TCR, T-cell receptor; UTR, Untranslated regions.
